# Blending Approach Preparation of PVA-*g*-PMA Films with Embedded “Green” Synthesized Silver Nanoparticles for Acetone Optical Detection

**DOI:** 10.3390/s23062941

**Published:** 2023-03-08

**Authors:** Katerina Lazarova, Darinka Christova, Daniela Karashanova, Biliana Georgieva, Gergana Marovska, Anton Slavov, Tsvetanka Babeva

**Affiliations:** 1 Institute of Optical Materials and Technologies “Acad. J. Malinowski”, Bulgarian Academy of Sciences, Akad. G. Bonchev Str., bl. 109, 1113 Sofia, Bulgaria; 2 Institute of Polymers, Bulgarian Academy of Sciences, Akad. G. Bonchev Str., bl. 103-A, 1113 Sofia, Bulgaria; 3 Department of Organic Chemistry and Inorganic Chemistry, Technological Faculty, University of Food Technologies, 26 Maritza Blvd, 4002 Plovdiv, Bulgaria

**Keywords:** optical sensor, acetone, thin films, polymers, Ag nanoparticles, silver, green synthesis, lavender

## Abstract

The blending approach (also known as the ex-situ approach) was used for the deposition of thin composite films comprising poly(vinyl alcohol-*graft*-methyl acrylate) (PVA-*g*-PMA) and silver nanoparticles (AgNPs). Firstly, the copolymer aqueous dispersion was synthesized through the redox polymerization of methyl acrylate (MA) on poly(vinyl alcohol) (PVA) using ammonium cerium (IV) nitrate as the initiator. Then, AgNPs were synthesized through a “green” method using the water extract of lavender based on by-products of the essential oil industry, and then they were blended with the polymer. Dynamic light scattering (DLS) and transmission electron microscopy (TEM) were used to determine nanoparticle size, along with their stability over time in suspension, during the 30-day period. Thin films of the PVA-*g*-PMA copolymer, with different AgNP volume fractions varying between 0.008 and 0.260%, were deposited via the spin-coating method on Si substrates, and their optical properties were explored. UV-VIS-NIR spectroscopy and non-linear curve fitting were used for the determination of the refractive index, extinction coefficient, and thickness of the films, while photoluminescence measurements at room temperature were conducted for studying the emission of the films. The concentration dependence of film thickness was observed and showed that thickness increased linearly from 31 nm to 75 nm when the nanoparticles’ weight content increased from 0.3 wt% to 2.3 wt%. The sensing properties toward acetone vapors were tested in a controlled atmosphere by measuring reflectance spectra before and during exposure to the analyte molecules in the same film spot; the swelling degree of films was calculated and compared to the corresponding undoped samples. It was shown that the concentration of AgNPs of 1.2 wt% in the films is optimal for the enhancement of the sensing response toward acetone. The influence of AgNPs on the films’ properties was revealed and discussed.

## 1. Introduction

In recent years, nanocomposite materials, composed of at least two materials that are combined to provide properties superior to those of the individual constituents, have been a subject of great interest and are widely studied. Especially of great interest is the use of composite materials in the development of sensors based on optical detection, where the response is determined by the characteristics of the individual components of the composite as well as the composite itself. Often, nanoparticles (NPs) of different types are incorporated into a material that is the main matrix. As the surface properties of the NPs are of great importance, the development of uniform, nanometer-sized particles has been extensively studied [1]. Attracting particular interest is the incorporation of nanoparticles in polymers, which has applications in diverse functional materials—thin optical films [2], photoswitchable devices [3], etc. With the continuous development of the field of nanotechnology, a wide variety of polymers have been used to improve their properties, with varying degrees of success, by using both ex-situ and in-situ methods to embed different types of metal nanoparticles [4]. In the case of the in-situ method, NPs are generated inside a polymer matrix through the chemical reduction of a metallic precursor or via diverse techniques, such as photolysis, radiolysis, or thermolysis [5]. When the ex-situ approach is used, already synthesized NPs are dispersed into polymeric matrices [6]. The incorporation of nanoparticles in the polymers leads to a change in their characteristics—free volume and swelling degree; crystallinity and glass-transition temperature, Tg; etc., thus providing the opportunity to control and tune the composite material properties [7]. Silver is one of the metal materials that exhibit antibacterial activity, chemical stability, the highest electrical and thermal conductivity, good light absorption, and high sensitivity. Therefore, they find applications across many fields, such as catalysis [8], comprising various electronic components [9], photonics and photography [10], biosensing applications [11] (because their surface plasmon resonance is strongly influenced by the surface-adsorbed molecules), etc. Also, the sensing ability of silver nanoparticles toward different analytes, such as glucose; phenol; triacylglyceride; H_2_O_2_; vanillin [12]; and heavy metals in water [13], including Hg^+^ [14], has been explored widely. 

Thus, hybrid polymer/metal nanoparticles materials [15] studies of the structure–property relationships are mostly needed for the progress in many areas, along with optical sensors, and due to the great demand for metal nanoparticles, many new eco-friendly, inexpensive methods for synthesis have been explored [16].

The development of a fully optical sensor based on color change from environmentally friendly materials is the prerequisite for finding the most suitable base material-dopant pairs. In recent years, many authors have confirmed the fact that green-synthesized NPs and, in particular, silver nanoparticles are potential candidates for biosensing and photocatalytic applications [17,18]. Different bioproducts were used as reductors for the synthesis due to their high contents of polyphenols and potent antioxidant capacities—including Buddleja globosa [19], the Hagenia abyssinica (Bruce) J.F. Gmel plant [20], and many various parts of plants, such as leaves, roots, flowers, fruits, rhizomes, etc. [21,22]. A very important asset of green synthesis is that there is no need to add stabilizing or capping agents to the nanoparticles, as some of the ingredients of the plant or bio extracts used play this role, thus reducing the degree of nanoparticle agglomeration. The comparison of sensitivity with the values reported in the literature shows that the green-synthesized AgNPs show very high sensitivity compared to particles synthesized via chemical reduction and pulsed laser deposition (PLD) methods; thus, they can be employed in various cost-effective and eco-friendly sensor device applications [17]. Nanoparticles synthesized via the green method have a number of advantages over the chemical, physical, and microbial methods, as it is a cheap, eco-friendly, one-step method, and does not require high pressure, energy, temperature, or toxic chemicals for production [23,24]. Likewise, it can be easily scaled up for large-scale synthesis. The method also avoids a number of problems of other preparation routes; for example, chemical synthesis, namely, has issues of high cost and short longevity of the particles, which is due to aggregation.

In most application cases, nanoparticle incorporation in the material provides the opportunity for refractive index tuning [25] and leads to the enhancement of the sensing response of the thin films used as sensing elements for different sensors [26]. The development of low-cost sensors that utilize simple detection principles and are fabricated from bio- and non-toxic materials will be a great advantage. In our previous work, we have shown that thin films of poly(vinyl alcohol-*graft*-methyl acrylate) (PVA-*g*-PMA) are suitable for the optical sensing of acetone vapors [27]. Polymers comprise environmentally friendly waterborne colloidal polymer particles that were spin-coated on non-porous substrates in order to make films with superior optical quality [27]. 

In this work, we focus on further enhancement of the performance of poly(vinyl alcohol-*graft*-methyl acrylate) (PVA-*g*-PMA) copolymer thin films. A blending approach was used for depositing composite thin films with various concentrations of biosynthesized “green” silver nanoparticles (AgNPs). AgNPs were obtained through the reduction of Ag^+^ with the water extract of lavender by-products from the essential oil industry. Then, they were incorporated into the copolymer’s matrix via an ex-situ approach. The characteristics of the particles and the composite thin film material were investigated. The impact of AgNPs on the film’s optical properties and optical response toward acetone vapors were studied and discussed.

## 2. Materials and Methods

### 2.1. Synthesis of Copolymer

The aqueous copolymer dispersion of PVA-*g*-PMA used in this work was synthesized through the redox polymerization of MA on PVA as initiating hydroxyl moiety, according to the procedures described in [27]. Briefly, a dilute solution of PVA (Sigma-Aldrich, St. Louis, MO, USA; Mw 9000–10000; 80% hydrolyzed) was prepared and bubbled with nitrogen for 15 min for oxygen removal. The solution was cooled down to 10 °C; then, the chosen amount of the initiator ammonium cerium(IV) nitrate (Sigma-Aldrich; ≥98.5%), dissolved in 1 N HNO_3_, was added under vigorous stirring, followed by the comonomer MA. The reaction mixture was bubbled with nitrogen for another 15 min and transferred to a bath thermostated at 35 °C. The polymerization was carried out for 3 h at 35 °C under a nitrogen atmosphere. The resulting PVA-*g*-PMA colloidal aqueous dispersion was purified by dialysis against deionized water and used for thin film deposition without additional treatment. A copolymer composition, analyzed using ^1^H NMR, was estimated as PVA_0.4_-g-PMA_0.6_, where the indexes denote the mole fractions of the components. Dynamic light scattering measurements of the obtained colloidal revealed average particle diameter DH = 73 nm and dispersity 0.067, as calculated by the formula described in [27].

### 2.2. Silver Nanoparticles Synthesis Method

#### 2.2.1. Preparation of Extracts from Lavender Waste

Lavender water extract was produced on the basis of *Lavandula angustifolia Mill.* solid by-products, received after plant essential oil steam distillation in Bul Phyto Oils Plc distillery (Zelenikovo, region of Plovdiv, Bulgaria, harvest 2018). The detailed recipe of this procedure, with the exact component ratios and time schedules, is described by Y. Lazarova et al. in [28]. The chemical composition of the extract is very complex and comprises various types of compounds, such as polyphenols, neutral sugars, soluble pectic polysaccharides, organic acids, alcohols, terpenes, esters, etc. For the electron transfer and Ag^+^ to Ag^0^ reduction, the most important are polyphenols, flavonoids, and low-molecular neutral sugars, with contents of 1469.1 ± 22.0 mg/L (total polyphenols), 318.87 ± 5.15 mg/L and 1107.91 ± 19.47 mg/L of extract, respectively.

#### 2.2.2. “Green” Synthesis of AgNPs

AgNPs were grown after the reduction of a silver salt—0.01 M AgNO_3_—aqueous solution using the water extract of lavender by-products, formation of seeds, and subsequent growth by their coalescence. First, 0.4 mL of water extract was mixed with the same quantity of distilled water, and 1.2 mL AgNO_3_ was added to this content. The color of the mixture was light yellow at the beginning, as this is the color of the diluted lavender extract and the color of AgNO_3_ is white; however, at the 15th minute after the mixture was prepared, the color started to darken, which is the indication of nanoparticle formation.

### 2.3. Determination of AgNPs Size, Morphology, Structure, and Concentration

The dynamic light scattering method (DLS) (ZetasizerNano ZS, Malvern, UK) was used for the measurement of the AgNPs size distribution profile in the suspension. For DLS analysis, 30 μL of the AgNP suspension was diluted in 1 mL of deionized water, and for every analysis, three measurements were conducted at room temperature (22 °C).

The AgNPs’ morphology, as well as the particles’ distribution in the polymer, were studied by transmission electron microscopy (TEM) using a high-resolution transmission electron microscope JEOL JEM 2100 (JEOL Ltd., Tokyo, Japan). For this purpose, drops of the AgNP suspension at different stages (15 min, 20 h, and 120 h) after mixing the Ag salt and the lavender extract were fixed on the standard TEM copper grids covered with amorphous carbon membranes and left for 24 h to dry in low humidity and dust-free ambiance. The same preparation procedure for TEM analysis was applied to the polymer with a 20 h AgNP sample.

A statistical determination of the AgNPs’ mean size and size distribution was performed on the basis of the TEM micrographs by measuring the diameters of a large number of individual particles with the freeware Image J [29] and presenting the corresponding histogram. The microstructure and the phase composition of the particles were studied by the diffraction modes of the microscope—selected area electron diffraction (SAED) and high-resolution TEM (HRTEM). The concentration of the silver in suspension was measured by inductively coupled plasma mass spectrometry (ICP-MS) as an analytical method based on the ionization of a sample by an extremely hot plasma made from argon gas in a 40.68 MHz ICP JY ULTIMA 2 spectrometer (Jobin Yvon, Longjumeau, France). The calculated value of the AgNP concentration was 80 mg/L. This value was further used to calculate the volume fraction of AgNPs in the composite films, as explained in detail in Section 2.4.

### 2.4. Composites Thin Film Preparation and Characterization

For the preparation of AgNP-doped films, an ex-situ approach was used. Firstly, AgNPs were synthesized using the “green” method described in Section 2.2.1 and Section 2.2.2, and then they were mixed with copolymer colloids at different weight ratios to polymer, ranging from 0.07 to 2.30 wt% (Table 1). Since the polymer and AgNP concentrations were known, 2.7 wt% and 80 mg/L, respectively, it was easy to calculate the volume fraction of silver nanoparticles in the films using Equation (1):

(1) $$f_{Ag}\left(\%\right)=100\frac{C_{m}}{C_{m}+\frac{\rho_{Ag}}{\rho_{p}}}$$

where *C_m_* = *m_Ag_*/*m_p_* is the mass ratio of AgNPs to polymer in the films and *⍴_Ag_* = 10.5 g·cm^−3^ and *⍴_p_* = 1.19 g·cm^−3^ are the density of the AgNPs and polymer, respectively.

Thin films of the AgNPs-doped copolymers (PAgx, x = 1–6) were deposited by spin-coating (WS-650-23 B, Laurell, North Wales, USA) 0.200 mL of the pre-mixed copolymer solution with AgNP suspension on pre-cleaned Si substrates at a rotation rate of 4000 rpm for 60 s. To ensure the complete drying of the films after deposition, they were annealed in air at 60 °C for 30 min. In order to study the impact of AgNPs on optical properties and the sensing response of the films, and to fully eliminate the possible influence of film thickness on these properties, pure copolymer films with the same thickness as the doped ones were prepared, as well, and are referred to as “corresponding polymer films”.

In order to determine the refractive index, n; extinction coefficient, k; and thickness, d, of the films, reflectance spectra, R, were measured at normal light incidence with a UV-Vis-NIR spectrophotometer (Cary 5E, Varian, Sydney, Australia) having an accuracy of 0.3%. The non-linear curve-fitting method, described elsewhere [30], was used to calculate n, k, and d from the measured spectra. The photoluminescence (PL) emission spectra of the composite films and the corresponding pure polymer films were measured at room temperature and an excitation wavelength of 290 nm with a FluoroLog3-22 spectrofluorometer (Horiba JobinYvon, Kyoto, Japan) equipped with a 450 W Xenon Lamp and used as an excitation source.

### 2.5. Sensing Experiments

**Figure 1:**
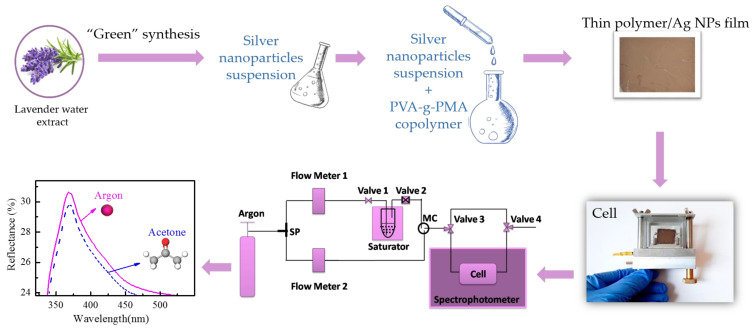
Scheme of the study process of the sensing properties of thin composite films with different concentrations of silver nanoparticles.

Acetone vapors were used to test the sensing ability of the thin films. The experiments were carried out by setting thin film samples into a quartz cell with an atmosphere control option via a homemade bubbler system connected to the cell [31]. The cell with the sample was placed in the spectrophotometer compartment, and measurements of the reflectance in different ambient surrounding -air, argon, and acetone, vapors were carried out without disturbing the sample, thus ensuring measurements at the same position of the sample. Reflectance spectra were further used for the calculation of n, k, and d in argon and acetone vapors. As the change of the atmosphere in the cell leads to a change and shift of the spectrum, the maximum reflectance change, ∆R, and swelling degree, SD, were calculated using Equations (2) and (3):

(2) $$∆R=\left|R_{ac}-R_{ar}\right|$$

(3) $$SD\left(\%\right)=100\left(d_{ac}-d_{ar}\right)/d$$

where *R_ac_* and *R_ar_* are the reflectance of the films in acetone and argon ambient vapors, and *d_ac_* and *d_ar_* are the respective thicknesses of the films. The whole process is illustrated in Figure 1. Argon was selected as a carrier gas because it is very dry and, in this way, the cross-sensitivity due to the presence of humidity was overcome. 

## 3. Results and Discussion

### 3.1. Characterization of Ag Nanoparticles

As the first step of our investigation, we studied the size and size distribution of Ag nanoparticles at different stages of their synthesis in order to find the time after which the rate of size change was very low.

#### 3.1.1. Size Investigation Using DLS

**Figure 2:**
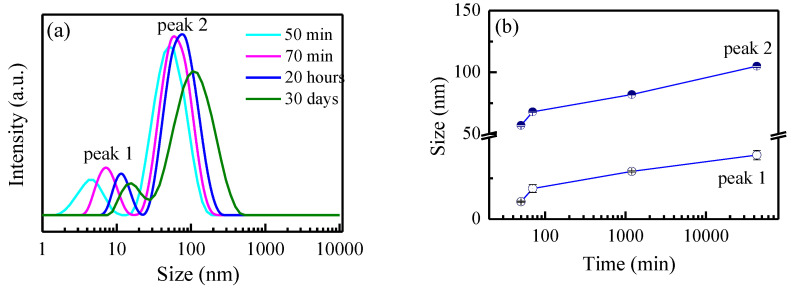
DLS curves for the size distribution of the silver nanoparticles at different times (noted in the graph) after the synthesis started (**a**). The dynamic of temporal change of silver nanoparticle size and corresponding root mean square errors (RMSEs), determined from DLS measurements (**b**).

Particle size and size distribution were determined via DLS measurements at different time intervals after the start of the synthesis: 50 min, 70 min, 20 h, and 30 days. The results obtained are shown in Figure 2a. 

As seen in Figure 2a, a bimodal distribution with two distinctive peaks was registered in all DLS curves, which is an indication that the AgNPs obtained could be divided into two populations by their sizes. According to the DLS measurement, 50 min after the beginning of the synthesis, the particles of the first population possess diameters in the range of 2–10 nm and an average size of 4.3 nm, and the particles of the second one showed a range of 15–150 nm with an average size of 57 nm. The presented distribution curves demonstrate a tendency to shift slightly toward higher particle diameters with time, and the values of the mean particle diameters became higher at the 20^th^ hour after the beginning of the synthesis compared to these at the shorter periods. This effect is well visualized in Figure 2b, which presents the dynamics of the temporal change of NPs’ sizes. It is seen that the trend for both populations is similar: a rapid change in the size is observed in the first stage of the synthesis and is most pronounced for the smaller particles. For a duration of 20 min, their size changes from 4.3 nm to 7.5 nm (i.e., the increase is more than 70%), while for the second population, the size changes from 57 nm to 68 nm, i.e., less than 20%. The growth rate decreases substantially in the next 20 h, and the size of the two NP populations reaches average values of 11.7 nm and 82 nm. During the next 30 days, the particles continue to grow very slowly and reach average values of 15.7 nm and 105 nm, respectively.

#### 3.1.2. TEM Investigation

**Figure 3:**
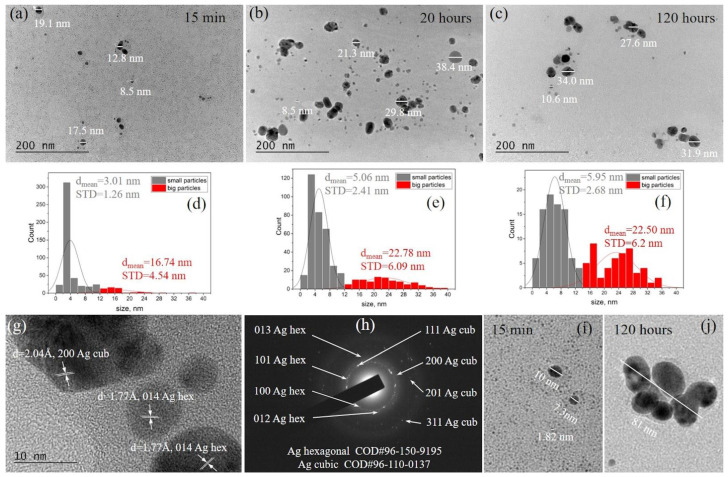
Bright Field TEM micrographs of the AgNPs 15 min (**a**), 20 h (**b**), and 120 h (**c**) after the beginning of the synthesis, and the corresponding histograms for 15 min (**d**), 20 h (**e**), and 120 h (**f**). The size distributions of the two particles’ populations are presented in different colors: grey for small particles and red for big particles. The representative HRTEM image (**g**) and SAED pattern (**h**) for the synthesized AgNPs revealed the phase composition of the particles. (**i**) and (**j**) represent cropped and zoomed BF TEM images visualizing seeds and small AgNPs and a cluster of AgNPs, respectively.

Visualization of the AgNPs, separated from the suspension and fixed on the TEM grid at the 15^th^ minute after the mixing of the components, was performed by Bright Field TEM and is presented in Figure 3a. The very tiny spherical particles of around 1–3 nm in diameter are homogeneously dispersed on the grid and illustrated at higher magnification in Figure 3i. At the early stage of the synthesis, these kinds of nanoparticles dominated in the suspension and served as seeds for the growth of larger AgNPs, some of which are also visualized on the same micrographs, and their diameters were evaluated to be in the range of 12–30 nm. It was established that, at the 15^th^ minute, the seeds represented 63% of the population of small nanoparticles, these ones with diameters in the range of 0–12 nm, as demonstrated in the histogram in Figure 3d. The presence of the tiny seeds determined the mean particle size of the first population at the 15^th^ minute to be equal to 3.01 nm with a standard deviation (STD) of 1.26 nm. After a 20 h stay in the suspension, a substantial change in the distribution of the diameters of the nanoparticles of the two populations was detected (Figure 3b,e). The seeds accounted for only 25% of the population of small particles, which predefined the mean size as 5.06 nm and STD = 2.41 nm. The large particles exhibited diameters from 12 nm to 40 nm, as presented in the histogram in Figure 3e, and their mean size was calculated as 22.78 nm (STD = 6.09 nm). The tendency for displacement toward higher particle size values with the time of the size distribution curves, both for small and large AgNPs, based on TEM measurements, coincided well with this one from DLS measurement. However, a difference was found in the values of the particle diameters determined by the two methods, due to the presence of organic shells on some particles, because of the action of some of the lavender extract constituents as a capping agent. Shells are visualized in the TEM micrographs in Figure 3a,i,j. They consist of light elements, predominantly carbon and nitrogen, and are electronically more transparent than AgNPs. This is why, in the statistical analysis of TEM images, only the diameters of AgNPs are included in the histograms, in contrast to the DLS measurements, where the sizes of the particles with their shells are taken into account. 

The differences in the data received from DLS and statistical analysis based on TEM are larger, especially for the population of bigger particles. DLS measurements show values at the maximum of peak 2 in Figure 2a, between 80 and 100 nm for the different periods after the beginning of the synthesis, which is in agreement with the results obtained by other authors studying the green synthesis of silver nanoparticles. Brajesh Kumar et al. reported the biosynthesis of silver nanoparticles using lavender leaf and the formation of spherical AgNPs of diameters in the range of 10–80 nm [18]. At the same time, for this population, the histograms (Figure 3d–f) demonstrate a size distribution in an interval between 12 and 40 nm. A detailed TEM study revealed that a limited number of individual Ag particles reached those large diameters of around 100 nm, but many clusters with this size appeared in the sample. Usually, they consisted of 4–6 AgNPs with diameters of about 10–40 nm, surrounded by a few small particles and altogether embedded in less electronic contrast material (probably organic ingredients) issued from the lavender extract. These kinds of clusters could be seen in Figure 3a–c, and one typical representative is illustrated in Figure 3j. 

The comparison with the TEM micrograph in Figure 3c for the particles, separated from the suspension at the 120^th^ hour, reveals that seeds and small particles still existed in the suspension and substantial changes in the diameters of the two particles populations were not registered, as also demonstrated in Figure 2b for DLS measurements. Logically, a decrease in the seeds’ number to 14% of the small particles’ population was found, as well as a decrease in the small particles’ number. 

A representative HRTEM image and SAED pattern of the AgNPs for all periods after the beginning of the synthesis are presented in Figure 3g,h, respectively. The measured interplanar distances from the HR micrograph and the diffraction pattern reveal the coexistence of the two phases of the Ag in the suspension: face-centered cubic Ag with the cell parameter a = 4.09 Å (Crystallography Open Database (COD) # 96-110-0137) and hexagonal Ag with the cell parameters a = 2.89 Å and c = 10.00 Å (COD # 96-150-9195).

**Figure 4:**
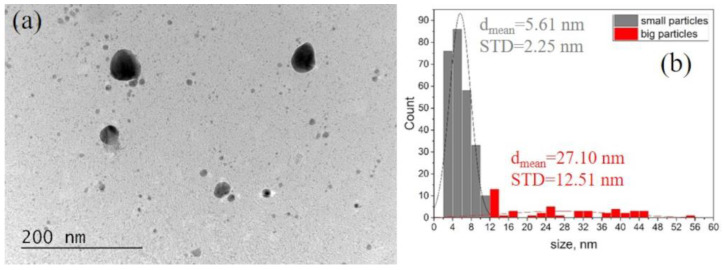
Representative Bright Field (BF) TEM micrograph (**a**) of the polymer/AgNPs film and the corresponding histogram of the size distribution of the 20 h AgNPs in the polymer (**b**).

The morphology of the composite polymer/AgNPs films was also followed by TEM. The silver nanoparticles were found to be relatively uniformly distributed in the polymer matrix at all silver concentrations. A representative BF TEM image is presented in Figure 4. Particles of different sizes are visualized to be present in the film (Figure 4a) and the histogram (Figure 4b). The size distribution of the AgNPs in the polymer revealed a mean size of 5.38 nm and an STD = 2.38 nm for the population of small particles and a mean size of 27.10 nm for the big ones, with an STD = 12.51 nm. It is seen that there is no agglomeration of the particles due to the blending with the polymer. The size distribution matches that of AgNPs in the suspension, presented in Figure 3e, indicating that the populations of AgNPs are not affected by the change in the medium from aqueous to a polymer.

### 3.2. Thin Films Characterization

As mentioned in Section 2.4. in order to determine the refractive index, n, and the extinction coefficient, k, along with the thickness, d, of the composite polymer/AgNPs films, reflectance spectra, R, of the films deposited on the silicon substrate were measured at the normal light incidence, and the non-linear curve-fitting method was used [30]. Table 2 shows calculated values of n, k, and d of composite films (subscript ‘c’) and corresponding polymers films (subscript ‘p’) and their nominal errors. It is seen from Table 2 that, for polymer films, there was no thickness dependence of the refractive index or extinction coefficient. Further, n and k for the Pagx (x = 1–6) films were not influenced by AgNP concentration. However, the concentration dependence of film thickness is observed: d of Pagx (x = 3–6) increases linearly from 31 nm to 75 nm when the AgNPs’ weight content increases from 0.28 wt% to 2.30 wt%.

The last column of Table 2 presents the expected increase in the refractive index of composite films compared to the undoped polymer due to the incorporation of AgNPs. The refractive index of composite films was calculated using Maxwell–Garnett effective medium approximation [32], assuming an effective medium consisting of two phases. The first one is a polymer matrix with a refractive index, np, and the second one is spherical non-interactive Ag nanoparticles with a bulk refractive index, nAg = 0.120 (600 nm) [33] and volume fractions calculated by Equation (1) and presented in Table 1. It is seen that the expected increase in n due to the addition of AgNPs is very small, and it is two orders of magnitude smaller than the experimental errors in refractive index determination. This result is expected considering the small volume fractions of the AgNPs in the composite films (Table 1). Therefore, a conclusion can be drawn that, within the studied thickness and concentration ranges, the refractive index and extinction coefficient of composite films and corresponding polymer films are independent of AgNPs concentration and film thickness.

**Figure 5:**
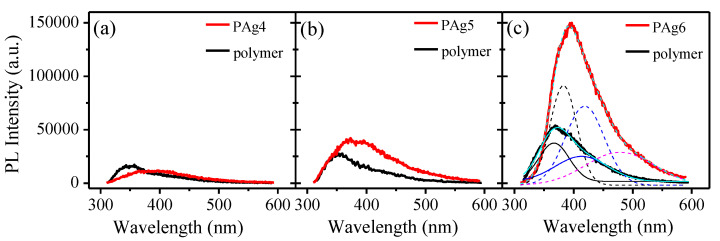
Photoluminescence emission spectra of Pag4 (**a**), Pag5 (**b**) and Pag6 (**c**) films and corresponding polymer films, measured at room temperature at an excitation wavelength of 290 nm.

In order to gain deeper insight into the optical behavior of polymer/AgNP films we studied their photoluminescence (PL) emission spectra. Figure 5 presents the PL emission spectra of Pagx (x = 4–6) and the corresponding pure polymer films measured at room temperature at an excitation wavelength of 290 nm. Both samples had emissions in the 300–600 nm spectral range, which is in accordance with the results of other authors [34,35]. An enhancement of PL emission intensity is observed with increasing film thickness for both doped and undoped films. The positive impact of AgNPs on the PL intensity of composite films is very well pronounced for films doped with 1.14 wt% and 2.30 wt% of AgNPs, Figure 5b,c, respectively. A threefold amplification of PL intensity is obtained when the polymer is doped with 2.30 wt% AgNPs. The possible explanation for the increased PL intensity is the surface-plasmon resonance in silver nanoparticles that induces strong local electric fields, thus enhancing the exciting and emitted photons [35].

A deconvolution of the PL emission spectra of the pure polymer film revealed two emission peaks centered at wavelengths of 367 nm and 413 nm. In the case of the doped film, both peaks shifted slightly toward higher wavelengths, 383 nm and 419 nm, respectively, and an additional wide emission peak, centered at 478 nm, appeared. Since this peak is not present in the PL spectra of a pure polymer film with the same thickness, we assigned it to the presence of Ag nanoparticles in the polymer. Similarly to the results obtained in [35], we attribute this peak to the radiative decay of surface plasmon resonance of silver nanoparticles. Our additional measurements of the transmittance of the films, not shown here, have confirmed the presence of an absorption band of the films around 450 nm. Therefore, emission centered at 478 nm is likely to be observed.

### 3.3. Sensing Properties of the Thin Films

**Figure 6:**
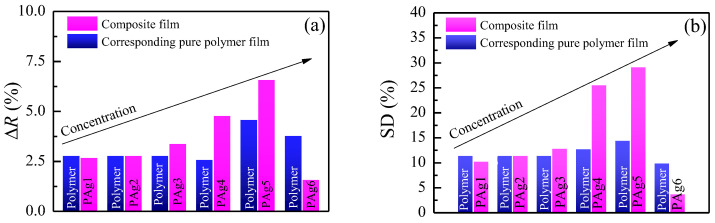
Reflectance change (**a**) and swelling degree (**b**) of PAgx films and corresponding polymer films due to exposure to acetone vapors.

In our previous studies, we have demonstrated that PVA-*g*-PMA films are sensitive toward acetone vapors and have a reproducible and reversible response [27]. When PVA-*g*-PMA films are exposed to acetone vapors, they absorb the vapors and swell. In order to evaluate the sensing ability of the thin composite films, the reflectance spectra of the samples were measured in the same spot prior to and during the acetone vapor exposure. The maximum reflectance difference for all samples is presented in Figure 6a. The measured spectra were further used for the calculation of n, k, and d in surrounding of argon and acetone vapors. The thickness change of films due to exposure to acetone vapors was used for the calculation of the swelling degree (SD (Equation (3)) and is plotted in Figure 6b. For low levels of doping (up to 0.3 wt%), there was no significant difference in the reflectance or swelling degree for both types of films. However, with an increase in the AgNP concentration, a substantial improvement of sensing response was observed: the swelling in the doped films reached values of 25% and 30% for Pag4 (0.57 wt% doping) and Pag5 (1.14 wt% doping) films, respectively; that is, 2 and 3 times more than the swelling of corresponding pure polymer films. It is interesting to note that a further increase of AgNP concentration to 2.3 wt% leads to the sharp deterioration of the sensing behavior of doped films: The swelling degree drops to 3.8%. 

Similar results of swelling enhancement in nanoparticle-doped photopolymers were obtained in [36,37,38]. The authors studied the formation of surface relief grating in a light-sensitive photopolymer and obtained a considerable increase of surface relief in photopolymers doped with Au nanoparticles with different sizes ranging from 10 to 50 nm [36] as compared to the undoped polymer. The authors explained the increase with the additional free volume introduced in the polymer matrix through embedding nanoparticles that facilitated the free movement of azo chromophores [36]. It is interesting to note that a similar enhancement was also obtained when a photopolymer was doped with ZnO NPs [37] or goethite (-FeOOH) nanorods [38]. Therefore, a conclusion can be drawn that the observed effect of swelling improvement is common and it is not inherent to a particular type of nanoparticle. Consequently, in our case, we may also assume that some amount of free volume is introduced in the PVA-g-PMA matrix when AgNPs are embedded inside. This increases, to some extent, the free space in the polymer matrix and facilitates the motion of polymer chains, thus increasing their ability to swell. It is seen from Figure 6b that there is an optimal concentration, over which the swelling degree significantly drops. The existence of an optimal concentration is in accordance with the results of other authors [36,37,38] and could be explained by the reinforcement of the polymer matrix due to the increased amount of AgNPs. When AgNP concentration increases, the polymer matrix becomes more rigid. This hinders the dimensional changes, thus overcoming the positive effect of the free volume introduction. 

## 4. Conclusions

The successful preparation and deposition of polymer/silver nanoparticle thin composite films through a blending approach were demonstrated. The films comprise a bio-compatible poly(vinyl alcohol-graft-methyl acrylate) matrix blended with ex-situ synthesized silver nanoparticles using a “green” method based on the water extract of lavender solid by-products (resulting from industrial steam distillation) as a reducing agent. It was revealed that the nanoparticles possess a bimodal size distribution and, at an early stage of the synthesis—up to one hour—very small AgNPs, with diameters of 1–3 nm dominate in the suspension. The majority of these nanoparticles serve as seeds for the growth of larger AgNPs in the two populations (0–12 nm and 12–40 nm in diameter, respectively), but some of them stay stable and represent a part of the suspension at the later periods studied. Clusters with an approximate size of 80–100 nm also existed. Every cluster consisted of 4–6 particles with diameters of about 10–40 nm each, usually surrounded by a few small particles and altogether enveloped in a tiny shell, most likely formed by the organic residue of lavender extract.

PL studies of the films at room temperature and an excitation wavelength of 290 nm demonstrated emission peaks in the 300–600 nm spectral range, with intensity increasing strongly with AgNP doping and slightly with film thickness. An optimal value of AgNP concentration around 1.2 wt% in the films was found, at which, the substantial enhancement of sensing responses toward acetone vapors was observed. It is assumed that extra free volume was introduced in the PVA-g-PMA matrix when AgNPs were embedded, which facilitated the swelling of polymer chains. Further increases in AgNP concentration resulted in a more rigid polymer matrix. This hindered the dimensional changes, thus overcoming the positive effect of the free volume introduction. 

## Figures and Tables

**Table 1:** Film code; weight ratio, *C_m_*, of AgNPs to polymer in wt%; and volume fraction, *f_Ag_*, of AgNPs in the films in %.

Film Code	C_m_ (wt%)	f_Ag_ (%)
PAg1	0.07	0.008
PAg2	0.14	0.016
PAg3	0.28	0.032
PAg4	0.57	0.065
PAg5	1.14	0.129
PAg6	2.30	0.260

**Table 2:** Refractive indices, *n_c_* and *n_p_*; extinction coefficients, *k_c_* and *k_p_*, at a wavelength of 600 nm; and thicknesses, *d_c_* and *d_p_*, in nm, of composite films (subscripts “*c*”) and corresponding pure polymer films (subscripts “*p*”). Δ*n_calc_* is the expected increase of *n_c_*, with respect to *n_p_*, due to the addition of AgNPs, calculated using the Maxwell–Garnett effective medium approach [32]. The last row presents the corresponding errors in the calculated parameters.

Sample	*n*_c_	*n*_p_	*k*_c_	*k*_p_	*d*_c_ (nm)	*d*_p_ (nm)	Δ*n*_calc_
PAg1	1.39	1.39	0.020	0.019	29	28	<10^−4^
PAg2	1.41	1.39	0.020	0.019	28	28	1 × 10^−4^
PAg3	1.42	1.39	0.020	0.019	31	28	1 × 10^−4^
PAg4	1.39	1.39	0.020	0.019	40	39	2 × 10^−4^
PAg5	1.37	1.36	0.020	0.019	48	48	5 × 10^−4^
PAg6	1.37	1.37	0.020	0.019	75	71	9 × 10^−4^
errors	±0.01	±0.01	±0.005	±0.005	±1	±1	-

## Data Availability

Not applicable.

## References

[1] Cattaneo S., Althahban S., Freakley S.J., Sankar M., Davies T., He Q., Dimitratos N., Kielyab C.J., Hutchings G.J. (2019). Synthesis of highly uniform and composition-controlled gold–palladium supported nanoparticles in continuous flow. Nanoscale.

[2] Yeshchenko O.A., Malynych S.Z., Polomarev S.O., Galabura Y., Chumanov G., Luzinov I. (2019). Towards sensor applications of a polymer/Ag nanoparticle nanocomposite film. RSC Adv..

[3] Pakula C., Zaporojtchenko V., Strunskus T., Zargarani D., Herges R., Faupel F. (2010). Reversible light-controlled conductance switching of azobenzene-based metal/polymer nanocomposites. Nanotechnology.

[4] Guo Q., Ghadiri R., Weigel T., Aumann A., Gurevich E.L., Esen C., Medenbach O., Cheng W., Chichkov B., Ostendorf A. (2014). Comparison of in situ and ex situ Methods for Synthesis of Two-Photon Polymerization Polymer Nanocomposites. Polymers.

[5] Waters W.A. (1984). Some Comments on the Development of Free Radical Chemistry. Notes Rec. R. Soc. Lond..

[6] Alonso A., Macanás J., Davies G., Gun’ko Y.K., Muñoz M., Muraviev D.N., Hashim A.A. (2011). Environmentally-Safe Polymer-Metal Nanocomposites with Most Favorable Distribution of Catalytically Active and Biocide Nanoparticles. Advances in Nanocomposite Technology.

[7] Kim Y.-G., Wichaita W., Thérien-Aubin H. (2020). Influence of the Architecture of Soft Polymer-Functionalized Polymer Nanoparticles on Their Dynamics in Suspension. Polymers.

[8] Bolla P.A., Huggias S., Serradell M.A., Ruggera J.F., Casella M.L. (2020). Synthesis and Catalytic Application of Silver Nanoparticles Supported on *Lactobacillus kefiri* S-Layer Proteins. Nanomaterials.

[9] Zhang J., Ahmadi M., Fargas G., Perinka N., Reguera J., Lanceros-Méndez S., Llanes L., Jiménez-Piqué E. (2022). Silver Nanoparticles for Conductive Inks: From Synthesis and Ink Formulation to Their Use in Printing Technologies. Metals.

[10] Caillosse E., Zaier M., Mezghani M., Hajjar-Garreau S., Vidal L., Lougnot D., Balan L. (2020). Photo-Induced Self-Assembly of Silver Nanoparticles for Rapid Generation of First and Second Surface Mirrors. ACS Appl. Nano Mater..

[11] Yu C.-X., Xiong F., Liu L.-L. (2020). Electrochemical Biosensors with Silver Nanoparticles as Signal Labels. Int. J. Electrochem..

[12] Dodevska T., Vasileva I., Denev P., Karashanova D., Georgieva B., Kovacheva D., Yantcheva N., Slavov A. (2019). Rosa damascena waste mediated synthesis of silver nanoparticles: Characteristics and application for an electrochemical sensing of hydrogen peroxide and vanillin. Mater. Chem. Phys..

[13] Prosposito P., Mochi F., Ciotta E., Casalboni M., De Matteis F., Venditti I., Fontana L., Testa G., Fratoddi I. (2016). Hydrophilic silver nanoparticles with tunable optical properties: Application for the detection of heavy metals in water. Beilstein J. Nanotechnol..

[14] Sanjeevappa H.K., Nilogal P., Rayaraddy R., Martis L.J., Osman S.M., Badiadka N., Yallappa S. (2022). Biosynthesized unmodified silver nanoparticles: A colorimetric optical sensor for detection of Hg^2+^ ions in aqueous solution. Results Chem..

[15] Venditti I. (2022). Metal Nanoparticles–Polymers Hybrid Materials I. Polymers.

[16] Bouafia A., Laouini S.E., Ahmed A.S.A., Soldatov A.V., Algarni H., Feng Chong K., Ali G.A.M. (2021). The Recent Progress on Silver Nanoparticles: Synthesis and Electronic Applications. Nanomaterials.

[17] Alex K.V., Pavai P.T., Rugmini R., Prasad M.S., Kamakshi K., Sekhar K.C. (2020). Green Synthesized Ag Nanoparticles for Bio-Sensing and Photocatalytic Applications. ACS Omega.

[18] Kumar B., Smita K., Cumbal L. (2016). Biosynthesis of silver nanoparticles using lavender leaf and their applications for catalytic, sensing, and antioxidant activities. Nanotechnol. Rev..

[19] Carmona E.R., Benito N., Plaza T., Recio-Sánchez G. (2017). Green synthesis of silver nanoparticles by using leaf extracts from the endemic Buddleja globosa hope. Green Chem. Lett. Rev..

[20] Melkamu W.W., Bitew L.T. (2021). Green synthesis of silver nanoparticles using *Hagenia abyssinica* (Bruce) J.F. Gmel plant leaf extract and their antibacterial and anti-oxidant activities. Heliyon.

[21] Vanlalveni C., Lallianrawna S., Biswas A., Selvaraj M., Changmai B., Rokhum S.L. (2021). Green synthesis of silver nanoparticles using plant extracts and their antimicrobial activities: A review of recent literature. RSC Adv..

[22] Salayová A., Bedlovičová Z., Daneu N., Baláž M., Bujňáková Z.B., Balážová L., Tkáčiková L. (2021). Green Synthesis of Silver Nanoparticles with Antibacterial Activity Using Various Medicinal Plant Extracts: Morphology and Antibacterial Efficacy. Nanomaterials.

[23] Hemmati H., Magnusson R. (2018). Development of tuned refractive-index nanocomposites to fabricate nanoimprinted optical devices. Opt. Mater. Express.

[24] Kulkarni A.G., Britto S.D., Jogaiah S., Jogaiah S., Singh H.B., Fraceto L.F., de Lima R. (2021). 18—Economic considerations and limitations of green synthesis vs. chemical synthesis of nanomaterials. Advances in Nano-Fertilizers and Nano-Pesticides in Agriculture.

[25] Ahmad H., Venugopal K., Rajagopal K., De Britto S., Nandini B., Pushpalatha H.G., Konappa N., Udayashankar A.C., Geetha N., Jogaiah S. (2020). Green Synthesis and Characterization of Zinc Oxide Nanoparticles Using *Eucalyptus globules* and Their Fungicidal Ability Against Pathogenic Fungi of Apple Orchards. Biomolecules.

[26] Sergeev A.A., Mironenko A.Y., Nazirov A.E., Leonov A.A., Voznesenskii S.S. (2017). Nanocomposite polymer structures for optical sensors of hydrogen sulfide. Technol. Phys..

[27] Bozhilova S., Lazarova K., Ivanova S., Karashanova D., Babeva T., Christova D. (2022). Colloidal Aqueous Dispersions of Methyl (meth)Acrylate-Grafted Polyvinyl Alcohol Designed for Thin Film Applications. Coatings.

[28] Lazarova Y.L., Dodevska T.M., Slavov A.M., Karashanova D.B., Georgieva B.C. (2019). Biosynthesized silver nanoparticles: Electrochemical application. Bulg. Chem. Com..

[29] ImageJ. https://imagej.net/ij/index.html.

[30] Lazarova K., Vasileva M., Marinov G., Babeva T. (2014). Optical characterization of sol–gel derived Nb_2_O_5_ thin films. Opt. Laser Technol..

[31] Lazarova K., Awala H., Thomas S., Vasileva M., Mintova S., Babeva T. (2014). Vapor Responsive One-Dimensional Photonic Crystals from Zeolite Nanoparticles and Metal Oxide Films for Optical Sensing. Sensors.

[32] Maxwell-Garnett J.C. (1904). Colours in metal glasses and in metallic films. Philos. Trans. R. Soc. Lond..

[33] Johnson P.B., Christy R.W. (1972). Optical Constants of the Noble Metals. Phys. Rev. B.

[34] Saini I., Rozra J., Chandak N., Aggarwal S., Sharma P.K., Sharma A. (2013). Tailoring of electrical, optical and structural properties of PVA by addition of Ag nanoparticles. Mater. Chem. Phys..

[35] Yeshchenko O.A., Dmitruk I.M., Alexeenko A.A., Losytskyy M.Y., Kotko A.V., Pinchuk A.O. (2009). Size-dependent surface-plasmon-enhanced photoluminescence from silver nanoparticles embedded in silica. Phys. Rev. B.

[36] Berberova-Buhova N., Nedelchev L., Mateev G., Stoykova E., Strijkova V., Nazarova D. (2021). Influence of the size of Au nanoparticles on the photoinduced birefringence and diffraction efficiency of polarization holographic gratings in thin films of azopolymer nanocomposites. Opt. Mater..

[37] Nedelchev L., Nazarova D., Dragostinova V., Karashanova D. (2012). Increase of photoinduced birefringence in a new type of anisotropic nanocomposite: Azopolymer doped with ZnO nanoparticles. Opt. Lett..

[38] Nedelchev L., Mateev G., Strijkova V., Salgueiriño V., Schmool D.S., Berberova-Buhova N., Stoykova E., Nazarova D. (2021). Tunable Polarization and Surface Relief Holographic Gratings in Azopolymer Nanocomposites with Incorporated Goethite (α-FeOOH) Nanorods. Photonics.

